# Mayo Clinic Infectious Diseases Board Review

**DOI:** 10.3201/eid1806.120388

**Published:** 2012-06

**Authors:** Sharon A. Bloom

**Affiliations:** Centers for Disease Control and Prevention, Atlanta, Georgia, USA

**Keywords:** infectious diseases, book review, synopsis, etiologic agents, major clinical syndromes, special hosts, special situations, Mayo Clinic

This 600-page board review book provides an excellent synopsis of infectious diseases. It is divided into 4 sections, each with a questions and answers section: General, Etiologic Agents, Select Major Clinical Syndromes, and Special Hosts and Situations. There are almost 200 questions in the book; however, they are not written in the American Board of Internal Medicine test format. Compared with the bulky binders filled with copies of electronic presentations that I took home from a live board review course, the Mayo Clinic book is a more useful and convenient desk reference.

The infectious disease information is presented in a way that facilitates understanding, not just memorization. Although organized differently, the content of the book is comparable to that presented in the live course. Some chapters were exceptional (e.g., Infections of the Central Nervous System, Obstetric and Gynecologic Issues Related to Infectious Diseases). I showed the book to an infectious disease fellow who passed the boards in 2011 with a score of 99%; that bright young fellow noted that the coverage of infectious diseases in transplant recipients and in patients with hematologic malignancies is “sketchy.”

In terms of practical examination preparation, the book is not as good as the live course when it comes to training test takers to recognize clues within the test. The book could more explicitly emphasize combinations of clues, or “buzz” words, that should trigger certain associations for the test taker (e.g., if question stem includes “cirrhosis and oysters,” look for “*Vibrio vulnificus*” among the answer choices). In addition, the book does not cover non–infectious disease syndromes masquerading as infections (e.g., rheumatologic syndromes), which, along with ethics, comprise up to 10% of the examination. The chapter on liver infections left me wanting more help distinguishing various types of viral hepatitis, i.e., hepatitis A–E, from other infectious causes of hepatitis, such as leptospirosis, as well as non-infectious causes of hepatitis. The book seems to have omitted certain important points, such as when vaccine against hepatitis A virus is recommended as a postexposure intervention.

In conclusion, the Mayo Clinic Infectious Diseases Board Review book is an excellent means to solidifying one’s infectious disease knowledge, and it could be a useful as an adjunct review tool. However, in terms of preparing for actual examination-style questions and for assuring that the full breadth of required information is covered, test takers will not want to rely on this book alone.

